# Application of Well Drainage on Treating Seepage-Induced Reservoir Landslides

**DOI:** 10.3390/ijerph17176030

**Published:** 2020-08-19

**Authors:** Zongxing Zou, Sha Lu, Fei Wang, Huiming Tang, Xinli Hu, Qinwen Tan, Yi Yuan

**Affiliations:** 1 Three Gorges Research Center for Geo-hazards, China University of Geosciences, Wuhan 430074, China; zouzongxing@cug.edu.cn (Z.Z.); wangfei_3762@cug.edu.cn (F.W.); tanghm@cug.edu.cn (H.T.); tanqinwen@cug.edu.cn (Q.T.); 2 School of Engineering, China University of Geosciences, Wuhan 430074, China; huxinli@cug.edu.cn; 3 Department of Land and Resources of Hubei Province, Wuhan 430074, China; yuany2019@126.com

**Keywords:** reservoir landslide, numerical simulation, landslide stability, Shuping landslide, drainage well

## Abstract

In the process of rapid drawdown of reservoir water level, the seepage force in the slide mass is an important factor for the stability reduction and deformation increment of many landslides in the reservoir areas. It is feasible to improve the stability of seepage-induced landslide by employing a drainage well to reduce or eliminate the water head difference that generates the seepage force. In this paper, the Shuping landslide, a typical seepage-induced landslide in the Three Gorges Reservoir area of China, is taken as an example. A series of numerical simulations were carried out to figure out the seepage field, and the Morgenstein–Price method was adopted to calculate the landslide stability. Then the influence of horizontal location of the drainage well, drainage well depth, drainage mode on the landslide treatment effect, and the applicability of drainage well were analyzed. The results show that: (1) landslide stability increases obviously with the well depth in the slide mass, while the increment of landslide stability with the well depth is limited in the slide bed; (2) the sensitivity of the stability improvement with the depth is greater than that with the horizontal positions of the drainage wells in the slide mass; (3) the drainage well is suggested to be operated when the reservoir water falls rather than operates all the time; and (4) the drainage method is most suitable for landslides with low and medium permeability. These results provide deep insights into the treatment of seepage-induced landslides.

## 1. Introduction

The continuous construction of hydropower projects around the world has been greatly changing the surrounding hydrogeological environments and triggering numerous geo-hazards. The most famous reservoir landslide is the 1963 Vajont landslide that occurred in Italy, resulting in the destruction of Longarone village and the loss of approximately 2000 lives [1]. Also well known, is the 2003 Qianjiangping landslide, which was triggered after the first trial storage of the Three Gorges Reservoir. This landslide ruined 346 houses, took 24 lives, and caused 1200 people homeless [2]. These famous disaster events occurred in reservoir areas resulted in severe consequences of casualties and economic losses, attracting wide public concerns.

In the normal operation of reservoirs, the storage can be quickly vacated in rainy seasons to prevent flooding, leading to rapid drawdown of reservoir water levels [3]. In this process, great seepage force pointing to the outside of landslide is produced, resulting in decreased stability and intensifying landslide deformation [4,5]. Nakamura [6] made statistics on reservoir landslides in Japan, and the results showed that about 60% of the reservoir landslides occurred during the water drawdown period. The seepage force induced landslides in the Three Gorges Reservoir area are widely distributed along the reservoir area, and 56% of the investigated landslides were deformed or slid in the period when reservoir water was dropping from high level to low level [7]. This type of landslide, induced by the outward seepage force during reservoir water drawdown, is identified as a seepage-induced landslide [8]. The prevention and control of seepage-induced landslides are of great significance in the mitigation of geological hazards in reservoir areas.

The reinforcement methods of reservoir landslides mainly include stabilizing piles, anchor cables, lattices, and retaining walls. Compared with other landslides, the effect of reservoir water is more emphasized in the study of reservoir landslide reinforcement. Numerical simulation results show that stabilizing piles and anchors have obvious anti-slide effects and have improved the stability of the Tanjiaping landslide in the Three Gorges Reservoir area during the period of water drawdown [9,10]. The thrust force on stabilizing piles was estimated with various theoretical methods by considering the impact of reservoir fluctuation [11,12]. Physical model tests were conducted on the landslides in the reservoir area to figure out the distribution characteristics of the pressure on stabilizing piles [13,14,15]. To further investigate the stabilizing characteristics of anti-slide piles under the reservoir operation condition, monitoring facilities were installed on testing piles of the Majiagou landslide in the Three Gorges Reservoir area [16,17]. Anchors and lattice structures are also commonly adopted to control reservoir landslides. Cantilever piles and anchors were installed in the Huangshibao landslide, which is a demonstration case in the Three Gorges Reservoir area [8].

Besides the reinforcement methods, drainage approaches are also applied to control reservoir landslides. Surface drainage can be found in almost every landslide control project. However, the fluctuation of reservoir water greatly affects the underground water level, and the effect of the surface drainage is limited in this situation. Thus, underground drainage is adopted to reduce the seepage force and control landslide motion. A drainage well is one underground drainage solution that reduces underground water levels and improves landslide stability [18,19,20]. The Brewery Creek landslide in New Zealand is controlled by deep drainage combined with tunnels and wells [21]. Huanglashi landslide is a case of draining control in the Three Gorges Reservoir area; drainage wells and adits are all applied [22,23].

At present, stabilizing piles, anchors, and other engineering structures are mainly used to prevent and control the seepage-induced landslides. However, large-scale landslides are developed with huge volume and depth, which bring great difficulties for implementing reinforcing structures. Underground drainage projects such as drainage wells are gradually applied in treating reservoir landslides, and this approach is particularly important and feasible for seepage-induced landslides. However, little research has been carried out to investigate the specific influencing factors and layouts of drainage wells in the treatment of seepage-induced landslides.

In this paper, the Shuping landslide in the Three Gorges Reservoir area of China is taken as the case to study the effects of horizontal locations of a drainage well, well depth, and drainage mode for controlling the effects of a seepage-induced landslide. The influence of different reservoir dynamic conditions and landslide permeability on selecting drainage wells are further revealed. The results provide a theoretical basis for reservoir landslide prevention and the design of drainage systems.

## 2. The Shuping Landslide—A Case of Seepage-Induced Landslide

**Figure 1:**
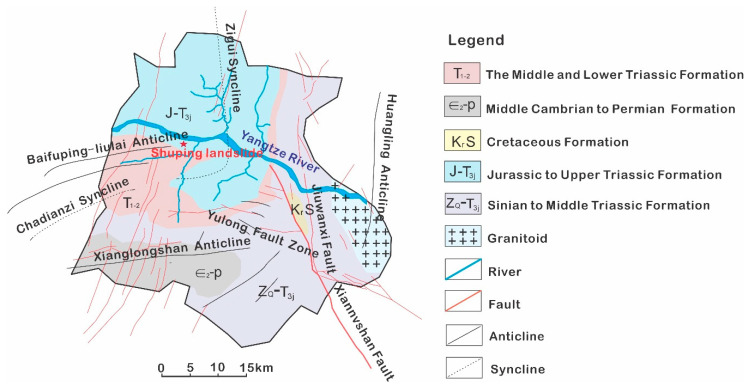
Geological map in the Shuping landslide area.

**Figure 2:**
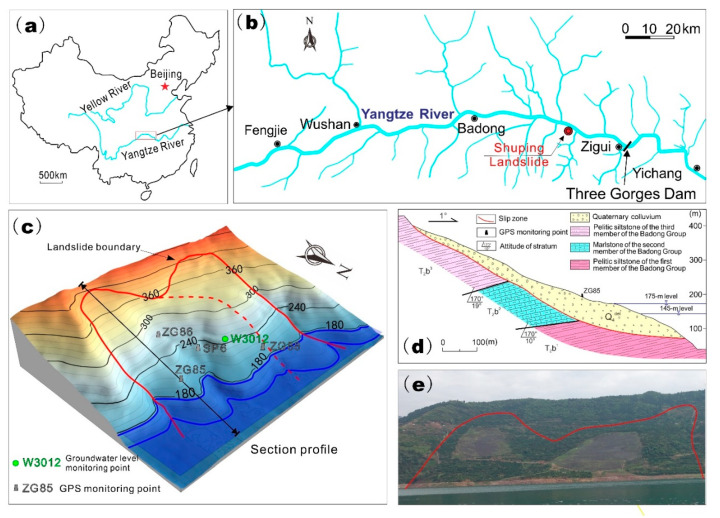
The location of the Shuping landslide (**a**,**b**). Plain view (**c**), with GPS monitoring points ZG85, ZG86, ZG88, SP6 and underground monitoring point W3012, and profile section (**d**) of the Shuping landslide. Full view of the Shuping landslide from the Yangtze River (**e**).

In Zigui county, faults, synclines, and anticlines develop (see Figure 1), affecting the formation of surrounding geo-hazards. The Shuping landslide is located in Zigui County of Hubei Province in China (Figure 2a) on the south bank of Yangtze River about 47 km upstream from the Three Gorges dam (Figure 2b). It was revived after the first impoundment of the Three Gorges Reservoir water in 2003. This landslide has a volume of approximately 2.7 million m^3^ and sits close to the main channel of the Yangtze River, posing a great threat to transportation on the Yangtze River [24,25]. Proper and effective measures are required to control this landslide.

### 2.1. Geological Condition

The Shuping landslide is a chair-shaped slope with dip angle of 20° to 30° toward the Yangtze River. The east and west boundaries of the Shuping landslide are characterized by gutters (Figure 2c,e). The elevation of the landslide ranges from about 400 m at the crown to 70 m at the toe. The front edge of the landslide is now submerged by the reservoir water. The slide mass has an average thickness of 50 m, with the length of 800 m in N-S and width of 700 m in W-E.

The Shuping landslide is seated on an anti-dip bedrock that is composed of marlstone and pelitic siltstone from the Triassic Badong Group (Figure 2d). This landslide can be divided into two parts as shown in Figure 2c. The east part deforms more seriously than the west part. The slide mass of the Shuping landslide is mainly composed of yellow and brown silty clay with blocks and gravel in the upper part, and dense clay and silty clay with gravel in the lower part. The slip zone is a 0.6–1.7 m thick layer consisting of yellowish–brown and steel gray silty clay extending along the surface of bedrocks.

### 2.2. In Situ Monitoring

The in situ monitoring layout in the Shuping landslide consists of four global positioning system (GPS) survey points, installed on the main sliding mass. Three of the GPS monitoring points (namely, ZG85, ZG86, and ZG88) were set in June 2003 and the remaining GPS monitoring point (SP6) was set in August 2007 (see Figure 2c for locations). All GPS monitoring points were surveyed every half month. In order to obtain the deformation data in real time, all GPS monitoring points were upgraded to remote automatic monitoring points on June 2012. The underground water level monitoring point (W3012) was constructed in November 2013 with a pneumatic water gauge. The orifice elevation of this point is 230 m above sea level. The underground water data were obtained in real time with remote automatic monitoring. The daily rainfall records were obtained from the meteorological station near the Shuping landslide. The daily reservoir level was measured upstream of the dam by China Three Gorges Corporation (source: http://www.ctg.com.cn/inc/sqsk.php).

### 2.3. Deformation Characteristics of the Shuping Landslide

**Figure 3:**
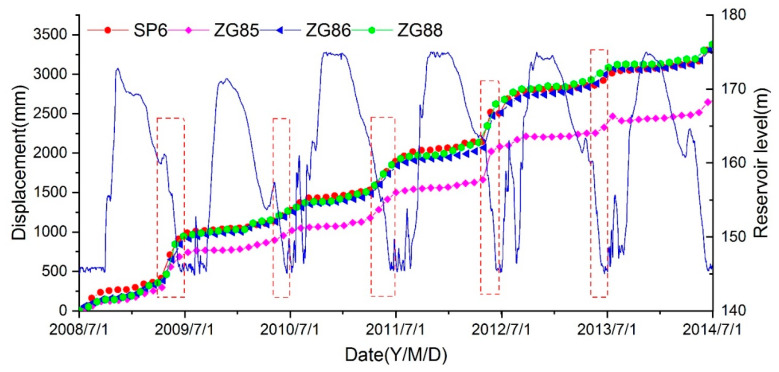
Monitoring data of the accumulative displacement and the reservoir water level of the Shuping landslide from July 2008 to July 2014. The red rectangular boxes represent the periods with faster movement.

Figure 3 presents the monitoring data of the Shuping landslide and reservoir level from July 2008 to July 2014 under annual fluctuations between 145 m and 175 m. The cumulative displacement curves display a step-like trend with a “jump” from April to June every year, when the reservoir water drops rapidly and stays at low level. However, the deformation is relatively small in other periods including the water rising period and the high level period. In 2009 and 2012, the deformation of the landslide exceeded 0.5 m during the rapid drawdown period, and two largest “jumps” were observed in these two years. In June 2012, a landslide warning was issued due to the rapid deformation rate.

**Figure 4:**
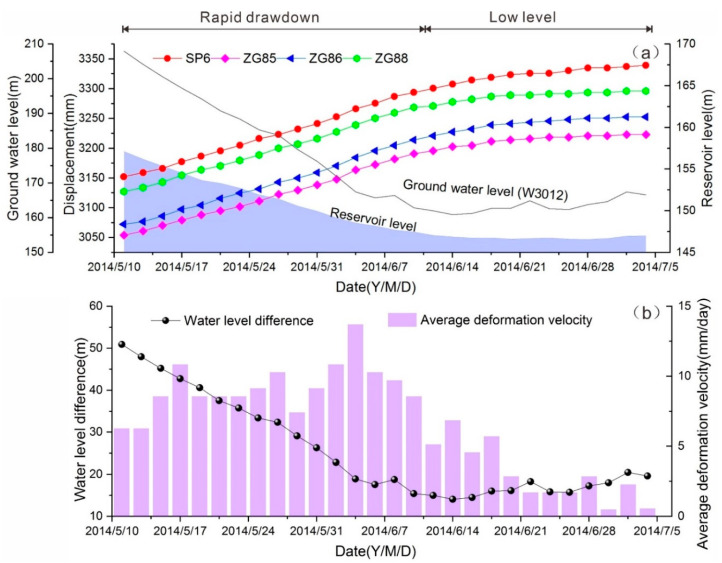
(**a**) Daily monitoring data of accumulative displacement and water level in the periods of rapid drawdown and low water level in 2014. (**b**) The relationship between the deformation velocity and the water level difference.

In order to further analyze the deformation mechanism of the Shuping landslide, the daily monitoring data of water level, groundwater level, and deformation of landslide during the period of rapid drawdown and low level during May to July of 2014 are presented. Figure 4a shows that the underground water level—monitored by station W3012 in the middle part of landslide—and landslide deformation was closely related to the variation of reservoir fluctuation. During the period of rapid drawdown of reservoir level, the monitored displacement of the landslide increased continuously. When the reservoir was running at a low level, the deformation of the landslide tended to be gentle. The relation curve between the water level difference (difference between underground water level and reservoir water level) and deformation rate of landslide is further shown in Figure 4b. It is indicated that the landslide deformation rate was high when the head difference was large, and the deformation rate then decreased as the head difference became stable. The high water head difference generated enormous outward seepage force, presenting a destabilizing effect on the landslide stability and thereby causing deformation in these periods. In general, the deformation of the Shuping landslide was mainly driven by the outward seepage force in the rapid drawdown period, making the Shuping landslide a typical seepage-induced landslide.

## 3. Methodology

As a typical seepage-induced landslide, the Shuping landslide is taken as a case in the numerical simulation of landslide treatment by well drainage. The seepage field of the landslide under the operation of drainage wells was calculated based on the saturated–unsaturated theory. Next, the stability of landslide was obtained by the Morgenstein–Price method [26] based on the obtained seepage field.

### 3.1. Saturated–Unsaturated Seepage Theory

In the process of reservoir water level fluctuation, the slope above the groundwater level is in unsaturated state, and the area below the reservoir water level is in saturated state. As the reservoir water level drops, the pore water pressure in the slope gradually dissipates, and the originally saturated soil becomes unsaturated. Therefore, in this study, the saturated–unsaturated theory was employed to calculate the slope seepage field under the conditions of reservoir level variation and draining from wells. When the total water head *H* is used as the dependent variable of the governing equation. The governing equation [27] for anisotropic two-dimensional saturated-unsaturated seepage is

(1) $$\frac{\partial }{\partial x}\left(k_{x}\frac{\partial H}{\partial x}\right)+\frac{\partial }{\partial y}\left(k_{y}\frac{\partial H}{\partial y}\right)=m_{w}\rho_{w}g\frac{\partial H}{\partial t}$$

where *k_x_* and *k_y_* are the hydraulic conductivities in the *x*-direction and *y*-direction, respectively; *m*_w_ is the specific water capacity, defined as the negative value of the partial derivative of the volume moisture content *θ*_w_ to the matric suction (*u*_a_ − *u*_w_), i.e., $m_{w}=-\frac{\partial \theta_{w}}{\partial (u_{a}-u_{w})}$; *u*_a_ and *u*_w_ are the pore-air pressure and pore-water pressure, respectively; *ρ*_w_ is the water density; *g* is the gravitational acceleration; and *t* is time.

The boundary conditions mainly include head boundary and discharge boundary, which can be written as below

Head boundary:

(2) $${k\frac{\partial H}{\partial n}|}_{\Gamma_{1}}=H\left(x,y,t\right)$$

Discharge boundary:

(3) $${k\frac{\partial H}{\partial n}|}_{\Gamma_{2}}=q\left(x,y,t\right)$$

where *k* is the hydraulic conductivity tensor, *n* is unit normal vector of boundary interface, and *q* is the discharge.

The saturated–unsaturated numerical calculation can be performed by the software package SLOPE/W module of GeoStudio2007 (Geo-Slope International Ltd., Calgary, AB, Canada 2007).

### 3.2. Numerical Model for the Shuping Landslide

**Figure 5:**
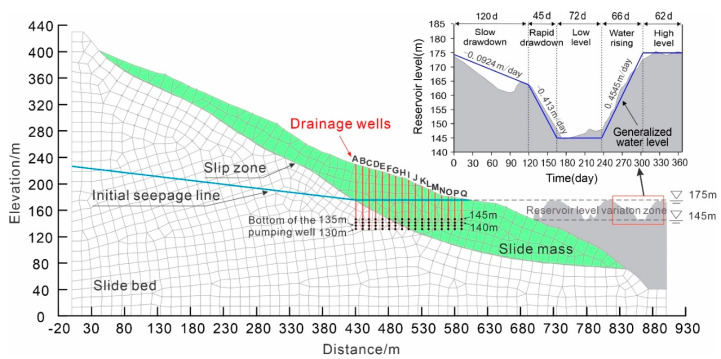
Numerical simulation model of the Shuping landslide.

Figure 5 shows the numerical simulation model of the Shuping landslide with the section profile from Figure 2. This numerical model had a length of 900 m and a maximum height of 430 m. The slide mass, slip zone, and slide bed are assigned with different material properties as shown in Table 1. The generalized model had a height of 379.5 m and length of 800 m, meshed into 6656 units with 6795 nodes for further calculation.

**Figure 6:**
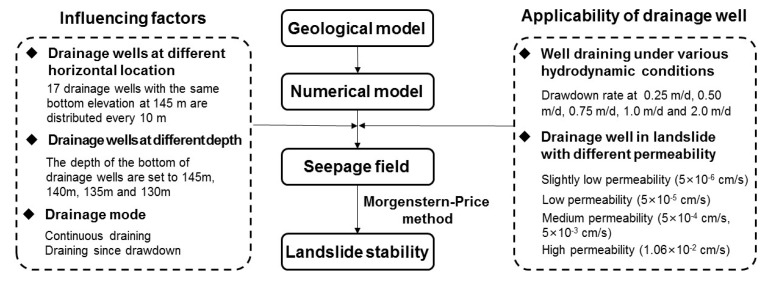
Framework of well drainage analysis.

Boundary conditions were set according to the features of the landslide. Zero flux boundary conditions were assigned along the bottom horizontal and right vertical boundaries. A series of trial modelling tests and in situ monitoring of water tables at W3012 were adopted to determine the constant water head at the left vertical boundary. The water head at the back boundary was 230 m from investigation material. The generalized hydrograph of the Three Gorges Reservoir (see Figure 6) was used to define the right boundary adjacent to the reservoir.

The simulation of drainage process can be realized by flow control or head control, corresponding to two different situations. Flow control is adopted when the process of water level change of the landslide is simulated under the condition of a known drainage capacity. Head control is adopted when the water level in the drainage well is constant at a certain depth. In the implementation of a drainage well, the required pumpage and corresponding pumping capacity need to be calculated and designed. Thus, the second situation is more suitable in practical engineering and the water head control method is adopted to simulate the well drainage in the Shuping landslide.

In the well drainage simulation, the size of the drainage well is much smaller than that of the landslide, therefore the method of simplifying wells to points can be adopted [28]. This method is easily accomplished in a simulation and the replacement of point instead of well has been a commonly used drainage simulation method. Point instead of well can be used to simulate a drainage well in a landslide with uniform permeability. However, if the bottom of the drainage well is located in the slide bed or the slide zone, the simulation results of the point instead of well method are not consistent with the actual situation [29]. The main reason is that the slide zone is a clay layer with lower permeability and the slide bed is a bedrock with lower permeability; it is difficult for the bottom of the well to construct a hydraulic connection with the slide mass in the simulation. In order to solve the above simulation problem of point instead of well in slide bed and slide zone with lower permeability, the area between the bottom of the well and the bottom of the slide mass is replaced by the slide mass permeability within a certain aperture.

As shown in Figure 5, the reservoir level periodically fluctuated between 145 m and 175 m, which can be divided into five phases including slow drawdown phase, rapid drawdown phase, low level phase, water rising phase, and high level phase. In the slow drawdown phase, usually from the beginning of January to the end of April, water drops from the highest level at a relatively low drawdown rate averaging 0.0924 m/day. In the consequent rapid drawdown phase (45 days duration on average), the reservoir drops at a high rate of about 0.413 m/day to attain its lowest level of 145 m in the middle of June. Then the reservoir is maintained at the lowest level for about 72 days. At the end of August, the reservoir manages to rise at an average rate of 0.4545 m/day, until it reaches the highest level of 175 m in about 66 days. In the subsequent and final phase, the water level is maintained at its highest annual level of 175 m for about three months. To simulate the seepage of the Shuping landslide and the drawdown effect on the landslide stability, the generalized reservoir level of slow drawdown, rapid drawdown, and the lowest water level were adopted as dynamic hydraulic boundaries (Figure 5). The initial seepage line is shown in Figure 5.

The stability of the landslide was evaluated by the factor of safety calculated with Morgenstern–Price method within SLOPE/W module of GeoStudio software (http://www.geoslope.com). In the stability calculation, slip zone material plays a key role and Mohr Coulomb model is selected for this material. The hydrological conditions are considered in the stability calculation by introducing the groundwater tables from the seepage simulation results within the SEEP/W module of the GeoStudio software. A series of numerical simulations were carried out to figure out the seepage field, and the Morgenstern–Price method was adopted to calculate the landslide stability. The main framework of this study is shown in Figure 6.

## 4. Analysis of Influencing Factors on the Treatment Effect of Drainage Wells

The stability of a landslide under a drainage treatment is affected by characteristics of a drainage well. In this section, the influence of horizontal location, depth of drainage well, and draining mode were analyzed through the simulation on the Shuping landslide under various draining conditions. In the whole analysis process, one drainage well was operating at a time to evaluate the treatment effect.

### 4.1. Horizontal Location of Drainage Wells

**Figure 7:**
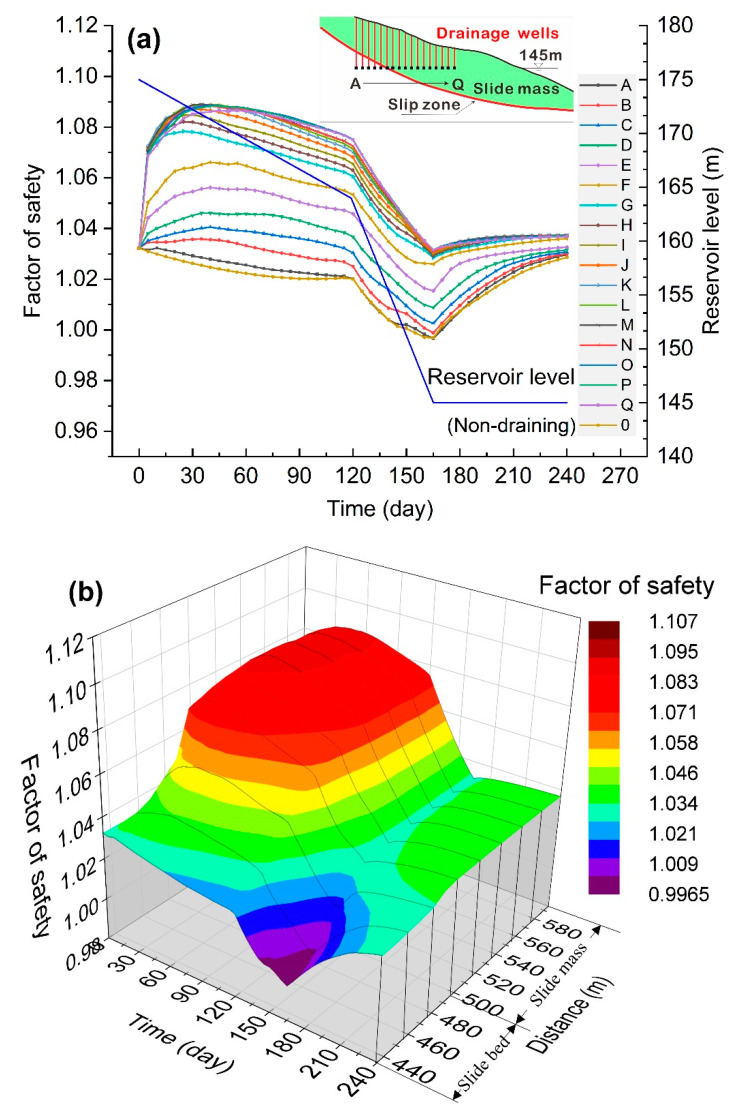
(**a**) Variation of the landslide stability for drainage wells at different horizontal locations; (**b**) cloud map showing how the landslide stability varies with the horizontal location and time.

Representative locations of drainage wells were designed according to the real hydraulic condition. Considering that the water level in the Three Gorges Reservoir area fluctuates between 145 and 175 m annually, the bottom depth of the drainage wells were set at the lowest elevation of the reservoir water level. The drainage well head in the side close to reservoir was arranged near the high water level of 175 m. Seventeen drainage wells with the same bottom elevation were distributed every 10 m from the reservoir side to the inner side of the slope, representing 17 draining conditions. The drainage wells from the inner side to the reservoir side are labelled A to Q (see Figure 5). Among these wells, six (A to F) were located in the slide bed or slip zone, and 11 (G to Q) were in the slide mass (see Figure 7a). For comparison, a non-draining condition was analyzed and labelled as 0 in the following discussions. The draining in a simulation model starts with the drawdown of reservoir water from the highest water level. At the same time, the stability of the Shuping landslide was analyzed under various draining conditions of different drainage well locations.

It is shown in Figure 7a that under the draining condition, the stability of the landslide was improved compared with the non-draining condition. As the drainage well position changes from well A at the inner side to well Q at the reservoir side of the slope, the stability of the landslide was gradually improved. The variation characteristics of the landslide stability share similar trends under the effects of different horizontal locations of drainage wells. When well A, the most inside well of the landslide, was under draining, the overall stability evolved similarly with that under the non-draining condition. As the location of the drainage wells extended from the inside of the slide bed to the outside, the stability of the landslide was steadily improved from well B to well F. As the wells in slide bed or slip zone operated during the beginning 15 days, the overall factor of safety of the landslide increased from the initial value to the highest value, while the increasing rate gradually decreased. As for well G to well Q, draining effects from slide mass, the factor of safety of the landslide increased rapidly in a straight line at the initial stage of draining as the water was drawn down in the first 10 days. With the further decline of reservoir water, the growth rate of safety factor gradually decreased, and the overall stability reached the maximum value at 30 days; then the landslide stability started declining as the water was drawn down.

Figure 7b shows obvious boundaries in the cloud map between wells in the slide bed (well A to well F) and in slide mass (well G to well Q), indicating that the well drainage method was more effective in the slide mass. For well G to well Q, the corresponding longitudinal location at 495 m to 595 m, the landslide stability obtained an obvious improvement in the initial draining stage. The stability received the most improvement at the longitudinal location at 540 m to 590 m, with nearly 6% increment. The factor of safety reached the lowest value when the reservoir level was 145 m. Figure 7a and b also shows that the increase of the lowest landslide stability with the well horizontal position in the slide mass is unobvious.

In conclusion, the operation of drainage wells improved the overall stability of the landslide. During the drawdown of reservoir water, the drainage from wells weakened the groundwater seepage effects, and the seepage force caused by the reservoir water level drop to reduce, leading to the improvement in landslide stability and the prevention of the real landslide condition. The drainage effects from well G to well Q in slide mass were more significant compared to wells A to F during the initial stage of reservoir water drawdown. Drainage from well A to well F were all located in the slide bed or slip zone area, accompanied with low water permeability. The function of the drainage wells could not effectively remove groundwater in the slide mass, therefore it is difficult to eliminate groundwater seepage force, leading to poor treatment effects in these areas. Due to a shorter seepage path and lower hysteresis of the draining effect of the drainage close to the reservoir side, it is more conducive to efficiently reduce the seepage force in the landslide.

**Figure 8:**
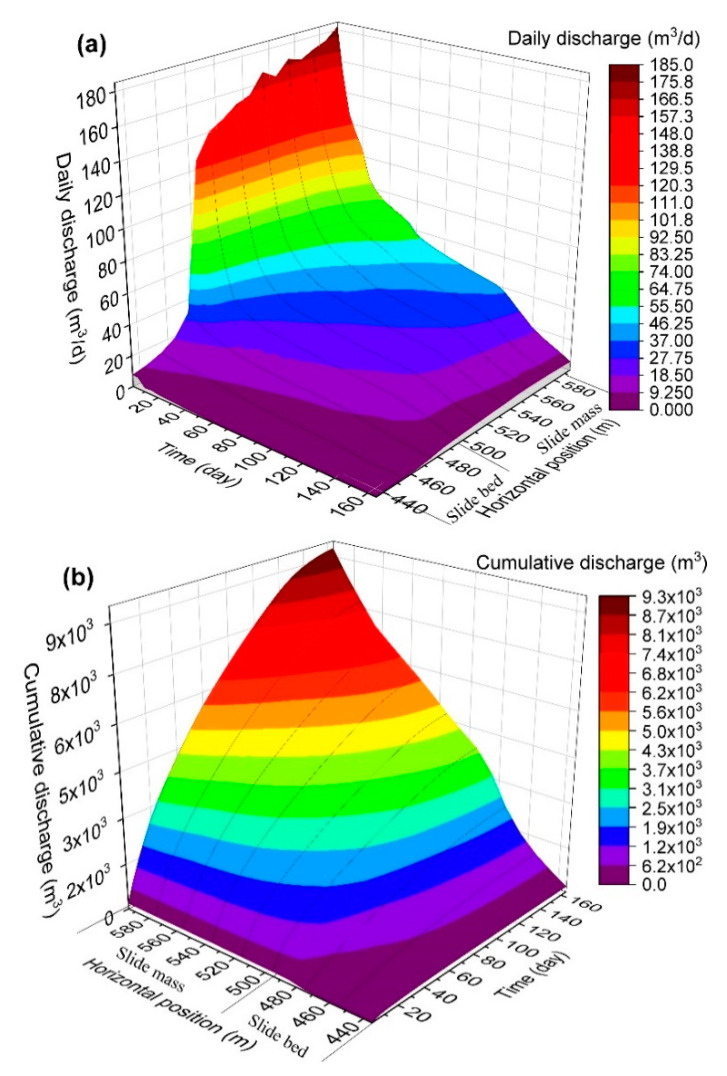
Cloud map showing the daily discharge (**a**) and cumulative discharge (**b**) with different draining well locations and time. The model is two dimensional and the discharges are in per-unit width.

**Figure 9:**
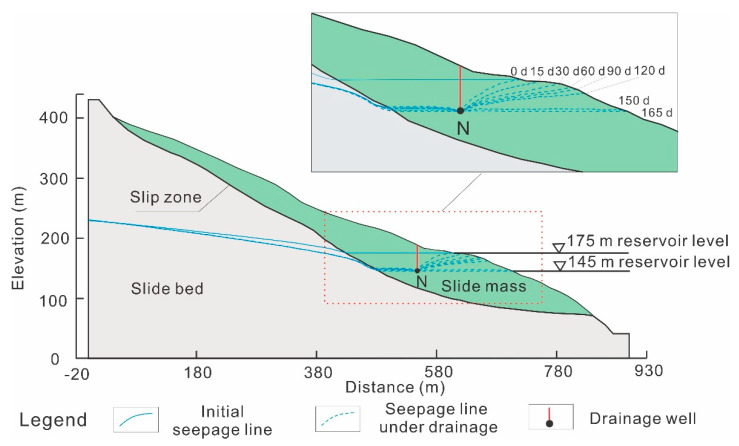
Phreatic lines in the process of reservoir level change combined with Drainage well N.

The overall trend in Figure 8a indicates that the daily discharge decreased with time while increased with horizontal positions. For wells in slide mass (well G to well Q which were from horizontal position 495 m to 595 m), the daily discharge was large in the first 15 days of drainage, followed by the formation of stable “landing funnel” shape (Figure 9) with a significant reduction of daily discharge. The daily discharge was basically stable from day 15 to 120. A further steep drop occurred between 120 to 165 days and the value of daily discharge gradually approached 0 after 165 days. This phenomenon indicates that in the process of reservoir water drawdown, the slide mass contains enough groundwater when the reservoir water is at high level at the initial stage, and it requires a large flow to reach the head at the well. As the water further declined, parts of the rock and soil mass of landslide turned from a saturated to an unsaturated state while the daily discharge was decreased to keep the water head in the same well. The daily discharge evolved correspondingly with the reservoir water drawdown. The change rules of the cumulative discharge in Figure 8b is consistent with that of the daily discharge in Figure 8a. The overall cumulative discharge of different positions of drainage wells changed similarly while the cumulative discharge at well Q near the reservoir side was 23 times of that from well F in the inner side, and the cumulative discharge was 51 times. This phenomenon indicates that in the early stage of water drawdown, the required discharge originates from the reservoir water, leading to the most cumulative discharge in this period.

In general, the landslide stability improved obviously under the draining effects of drainage wells in the slide mass. However, the improvement on landslide stability was limited in the front part of the slide mass compared with that in the inner part of slide mass. At the same time, the cumulative discharge grew along with the increase of horizontal position (close to the reservoir side). The horizontal position can be decided according to the pumping capacity of different well positions. In order to avoid large pumping flow, the horizontal position of pumping wells should be kept a distance from the reservoir. The layout of the drainage well should be located at the inner side in the front part of the landslide in order to ensure the treatment effect and improve the engineering efficiency. The layout of the horizontal position of the drainage well needs to comprehensively consider both the stability improvement effect and the discharge volume.

### 4.2. Depth of Drainage Wells

**Figure 10:**
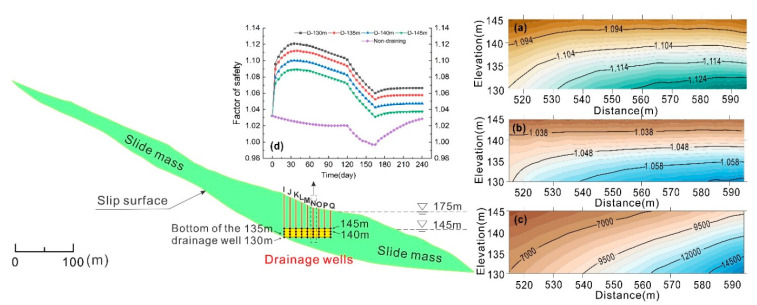
Variation of the landslide stability for draining wells at various depths. (**a**) Contour map of maximum safety factor during draining and reservoir level variation. (**b**) Contour map of minimum safety factor during draining and reservoir level variation. (**c**) Contour map of cumulative discharge during draining and reservoir level variation. (**d**) Variation of the landslide stability under well drainage at various depths with a horizontal location of 565 m.

Besides the horizontal locations of drainage wells, the vertical distribution (depth) also affects the seepage field after drainage, thus changing the landslide stability. Therefore, it is necessary to conduct quantitative analysis of the depth of drainage wells and the change in the groundwater seepage field. According to the fluctuation rules of reservoir water in the Three Gorges Reservoir area, in a hydrological year, the reservoir water level fluctuates between 175 m and 145 m. The depth of the bottom of the drainage wells were set to 145 m, 140 m, 135 m, and 130 m, and corresponding analyses were carried out to reveal the effects of well depth on the drainage effects in the slide mass at the front part of the Shuping landslide (Figure 10).

According to Figure 10a,b, the factor of safety of the landslide was affected by both the depth and horizontal location of drainage wells. On the whole, the pumping effects of the drainage wells in the front part of landslide were relatively better for improving the stability. The stability value changed more significantly with the increase of depth than the variation of horizontal positions. Under the condition of draining by the wells at a depth of 130 m, the maximum factor of safety increased 2.7% from the horizontal position from 515 m to 595 m. This rule also fits the minimum safety factor (Figure 10b), with the increment of 1.9% in the same range. In addition, for the 145 m depth, the value of factor of safety increased gradually from the inner side to the reservoir side. It is shown in Figure 10c that, in the process of water drawdown, the cumulative discharge of the drainage well was affected as much by the depth as the horizontal positions of the drainage wells.

To further study the influence of depth change on the stability of landslide, for the drainage well at the horizontal position of 565 m, the depth of 130 m, 135 m, 140 m, and 145 m were applied in the drainage simulation and the stability characteristics were analyzed in these four conditions. According to Figure 10d, the change rules of factor of safety in the same horizontal position was similar despite the variation in depth. In general, the deeper the drainage well bottom was (the lower the elevation was), the greater the overall factor of safety was, indicating better improvement of landslide stability. The stability obtained improvement in different stages of water drawdown, and the stability decreased with the decline of reservoir water level.

In conclusion, the depth of drainage well in slide mass had a significant influence on landslide stability under draining, and the factor of safety increased with the growth of depth value. The draining effect of wells in the deep part was controlled by the combination of depth and horizontal location. With the increase of depth and horizontal position, the factor of safety and increment of cumulative discharge were both raised, with these results being more sensitive to the depth position of drainage wells than the horizontal position. The depth of drainage wells well had more influence than the horizontal position on landslide stability, and this phenomenon was more obvious at the location closer to the reservoir side.

### 4.3. Drainage Mode

**Figure 11:**
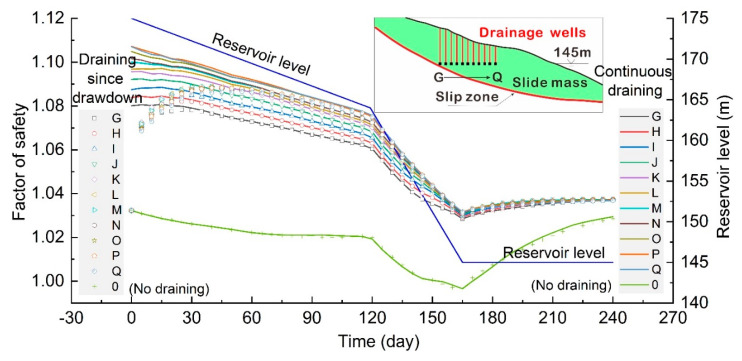
Comparison of the draining effect of different drainage modes on landslide treatment result.

In addition to drainage position, the mode deciding the draining time was also analyzed. In this study, two drainage modes were set, including continuous draining and draining since drawdown. Continuous draining is a kind of drainage mode whereby a drainage well operates all the time with water variations. Conversely, draining since drawdown is where a drainage well works only when the reservoir enters the stage of reservoir level decline. In order to compare and study the change rules of landslide stability under these two drainage modes, drainage well G to well Q with different horizontal positions with the same bottom elevation of 145 m (Figure 11) were arranged in the slide mass.

It is shown in Figure 11 that the difference of landslide stability under the conditions of continuous draining and draining since drawdown was mainly detected in the early stage. Two different drainage modes both significantly improved the stability of the landslide and achieve the purpose of landslide control. In the first two months, the stability of the landslide under the condition of continuous draining was greater than that under draining since drawdown condition. In the continuous draining situation, the seepage field of the slope reached a stable state in the high water period, and the safety factor of the landslide decreased gradually as the reservoir level declined.

In the draining since drawdown situation, the drainage well started working during the period of the reservoir level falling, and an inward seepage force that stabilized the slope was generated, and the landslide rapidly increased consequently. With time passing by, the difference between the landslide stability under the condition of continuous draining and draining since drawdown was smaller and smaller. After two months, the landslide stability of the two draining modes basically changed with the same trend before they got into a worse situation. Therefore, for the sake of economy, it is not necessary to keep continuously draining to improve the stability of the seepage-induced landslides.

## 5. Applicability of Drainage Well

The deep drainage technology was mainly developed to eliminate the seepage force in the slope, so as to improve the stability of landslides. The formation of seepage force in the slope of a reservoir is closely related to the hydrodynamic conditions of reservoir water fluctuations, such as the rate of water level variation, and the composition of landslide materials that determines the permeability. Therefore, it is necessary to analyze the applicability of drainage wells under different hydrodynamic conditions and material properties, so as to provide guidance for the decision of drainage applicability in seepage-induced landslides.

### 5.1. Applicability of Well Drainage under Various Hydrodynamic Conditions

In order to meet the dual functional requirements of reservoir flood control and power generation, the reservoir water fluctuates annually and become an important factor affecting the deformation and stability of landslides. The drawdown rate of water level has an important influence on the evolution of seepage-induced landslide. Based on the actual situation of water fluctuation in the Three Gorges Reservoir area, the simulated water level dropped from 175 m to 145 m at the rate of 0.25 m/d, 0.50 m/d, 0.75 m/d, 1.0 m/d, and 2.0 m/d, respectively, to simulate and analyze the impact of drawdown rate of reservoir level on the landslide stability. Drainage wells were set at different horizontal positions with the same elevation of 145 m.

**Figure 12:**
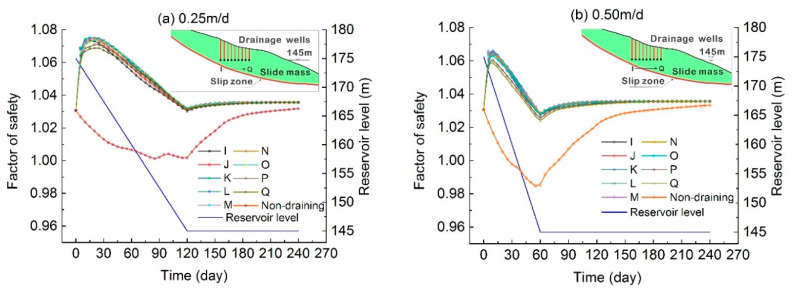

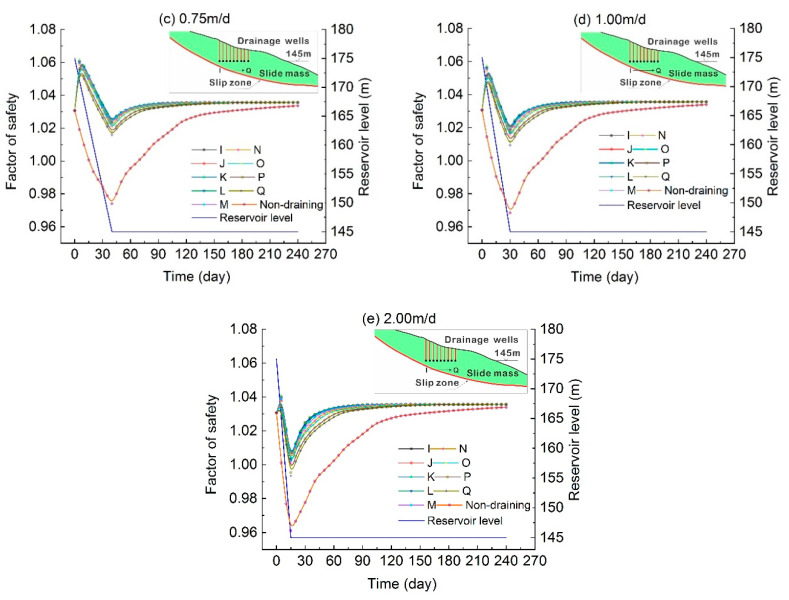
Variation of the landslide stability under different drawdown rates of the reservoir, with (**a**–**e**) refer to the condition with rate of 0.25 m/d, 0.50 m/d, 0.75 m/d, 1.00 m/d and 2.00 m/d, respectively.

Figure 12 shows the change curves of landslide stability under different conditions with various drainage wells at the elevation of 145 m every 10 m from the middle rear of slide mass to the front edge of slide mass, corresponding to the X-axis from 515 m to 595 m. Compared with the simulation results of each group, it can be found that the landslide stability decreased with the increase of the drawdown rate of the reservoir level. The most dangerous state occurred at the 145 m water level at the end of the drawdown stage of the reservoir level. As indicated in Figure 12, the landslide stability improved by setting deep drainage wells under various conditions of drawdown rate of reservoir water level.

The effect of well drainage on the landslide stability varied under different drawdown rates. When the reservoir was in the rapid drawdown stage (with the drawdown rate of 2 m/d), the drainage well mainly played a role in restraining the decline of landslide stability as the reservoir water drawdown, while in the process of relatively slow drawdown (with the drawdown rate of 0.25 m/d and 0.5 m/d), the overall landslide stability was improved significantly by well drainage compared with the state without drainage. When the drawdown rate of the water level was the same, the effect of draining on the stability of landslide with drainage wells at different positions in the slide mass was consistent during the whole process of reservoir water level decline. The stability was less sensitivity to the horizontal positions of the drainage well in the rapid drawdown condition, while in the slow drawdown condition, landslide stability was more sensitive to the horizontal location of drainage well resulting in a wider distribution of stability curves with different drainage wells (Figure 12). Overall, drainage well measures are feasible for improving the landslide stability under various hydrodynamic conditions of different drawdown rates.

### 5.2. Applicability of Drainage Well in Landslide with Different Permeability

The composition and structure of landslides in the reservoir area are quite different. Taking landslides in the Three Gorges Reservoir area as an example, some composition materials are composed of silty clay formed by the weathering argillaceous siltstone, while others are composed of loose gravelly soil formed by limestone, resulting in great difference in landslide permeability in the reservoir area. The statistical analysis of 369 landslides in the Three Gorges Reservoir area shows that the permeability coefficient of landslide ranges from 0.003 m/d and 9.2 m/d [30]. According to the code for water conservancy and hydropower engineering geological investigation [31], the permeability of rock and soil materials can be divided into six grades including extremely low permeability (K < 10^−6^ cm/s, equivalently, clay), slightly low permeability (10^−6^ cm/s ≤ K < 10^−5^ cm/s, equivalently, clay-silt), low permeability (10^−5^ cm/s ≤ K < 10^−4^ cm/s, equivalently, silt sand), medium permeability (10^−4^ cm/s ≤ K < 10^−2^ cm/s, equivalently, sand-gravel), high permeability (10^−2^ cm/s ≤ K < 1 cm/s, equivalently, sand gravel-block stone), and extremely high permeability (K > 1 cm/s, equivalently, block stone). According to the variation range of the permeability of the landslides in the Three Gorges Reservoir area and the classification standard of permeability of rock and soil, the landslide permeability selected for simulation was as follows: 5 × 10^−6^ cm/s was chosen for slightly low permeability; 5 × 10^−5^ cm/s was selected for low permeability; 5 × 10^−4^ cm/s and 5 × 10^−3^ cm/s were selected for medium permeability; the upper limit of permeability coefficient in reservoir area landslide 9.2 m/d (=1.06 × 10^−2^ cm/s) was selected for high permeability. A total of five permeabilities were selected representing four types of landslide with different permeability.

**Figure 13:**
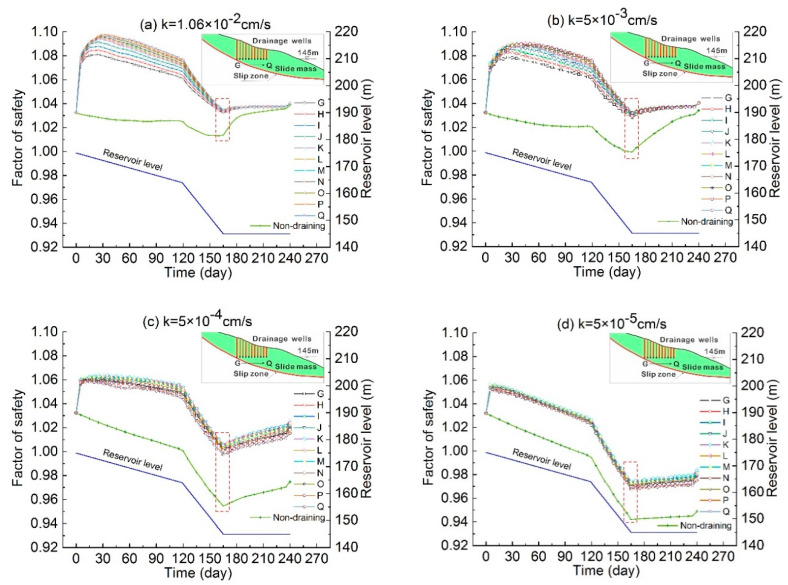

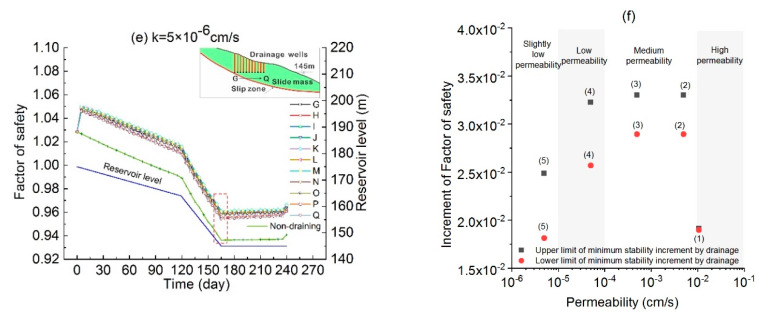
Variation of the stability of landslide with different permeabilities representing different material, under the condition with permeability (**a**) k = 1.06 × 10^−2^ cm/s; (**b**) k = 5 × 10^−3^ cm/s; (**c**) k = 5 × 10^−4^ cm/s; (**d**) k = 5 × 10^−5^ cm/s; (**e**) k = 5 × 10^−6^ cm/s. The relationship of increment of factor of safety and permeability are shown in (**f**). Stability of landslide of different permeabilities with the drainage wells at various positions (**a**–**e**). The increment of the minimum stability by comparing the drainage and non-draining conditions at the worst situation (**f**) as shown in the red rectangle in (**a**–**e**).

Figure 13 shows that when the drainage well was open during the drawdown period of reservoir, the landslide stability first increased then decreased with the reservoir drawdown, and obtained the minimum stability at the lowest reservoir level. The comparison results of landslide stability under well drainage and non-draining conditions show that the stability was improved by well drainage in the landslides with different permeabilities, but in different permeable landslides, the sensitivity of landslide stability to the horizontal positions of drainage wells was different. It demonstrates that the horizontal position of drainage well has a greater influence on the stability of a high permeability landslide than a lower permeability landslide. In addition, under the non-drainage condition, the stability of different permeable landslides decreased with the drawdown of the reservoir water level, and the stability of landslides with high permeability changed less, indicating that the change of reservoir water level has less impact on higher permeable landslides; the stability of lower permeable landslides decreased more, indicating that the drawdown of reservoir water level had a more significant impact on lower permeable landslides. The variation between the upper and lower limits was different due to different conditions, one in high water level and one in low water level.

The effect of drainage on the stability improvement varied from different permeable landslides. Under the drainage condition, the stability of the landslide with high permeability improved significantly more in the initial stage of reservoir decline, while the stability of the landslide with lower permeability was less improved. In the late stage of water level decline, the most dangerous state of landslide, the stability improvement evolved differently in landslides with different permeabilities. It is shown in Figure 13b that the most obvious stability improvement was in landslides with low permeability and medium permeability. In the landslides with low and medium permeability (represented by permeability coefficient of 5 × 10^−3^ cm/s, 5 × 10^−4^ cm/s and 5 × 10^−5^ cm/s), the factor of safety of the worst situation for various drainage wells was increased by 0.025–0.325.

## 6. Discussion

The plane position and depth of drainage wells were the key technical parameters for landslide control. Due to the complex composition of landslide masses, the physical and mechanical parameters of a landslide, including the permeability coefficient of a landslide, have strong heterogeneity [32]. It is difficult to determine the optimal horizontal position and depth of drainage wells using an analytical solution, but it can be solved by some mature intelligent optimization algorithms. The specific ideas are as follows: (1) according to the actual geological structure, the precise numerical model of landslide is established; (2) the horizontal position and depth of drainage wells are arranged with certain horizontal and vertical spacing; (3) the landslide stabilities under the condition of various positions and depths of drainage wells are calculated respectively; (4) based on the simulation data, support vector machine (SVM) or particle swarm optimization (PSO) and other intelligent algorithms [33] can be used to accurately solve the horizontal position and depth of the pumping well where the improvement of the stability coefficient is most obvious.

In landslides with low and medium permeabilities, drainage wells display better performance in the improvement of landslide stability than that with high and lower permeability. In high permeable landslides, seepage force is hard to generate when the reservoir level drops, indicating that the stability is less affected by the drop of reservoir level. In landslides with lower permeability, drainage wells cannot easily vacate the groundwater in the landslide, weakening drainage effects. Therefore, the stability improvement of deep drainage measures is most suitable in landslides with low and medium permeabilities.

## 7. Conclusions

Based on the monitoring data of the Shuping landslide, the basic characteristics of deformation and the effect of water level variation on the Shuping landslide were analyzed. A typical numerical model was established according to the geological structure model of the Shuping landslide and the operation characteristics of the Three Gorges Reservoir. The influencing factors of the effect of drainage wells on landslides were investigated. The following conclusions were reached:

(1) The stability of the landslide under the drainage condition was better than that without draining, which demonstrates that well drainage is one feasible way to control a seepage-induced landslide.

(2) In the slide mass, the sensitivity of the landslide stability to the depth of the drainage well was greater than that to the horizontal position of the drainage well, indicating that the stability was controlled significantly more by the depth of the drainage well. The horizontal position can be decided according to the pumping capacity of different well positions. In order to avoid large pumping flow, the horizontal position of a pumping well should be kept a distance from the reservoir.

(3) Due to the relatively low permeability coefficient of the slide bed, the increase of landslide stability under the effect of drainage well in the slide bed is not obvious. When the horizontal location of drainage well increased from the slide bed to slip mass, the improvement of landslide stability was more obvious. Therefore, drainage wells should be arranged in the slide mass. 

(4) Well drainages are most effective for the stability improvement of a landslide with low and medium permeabilities. It is not easy to generate seepage force in the slope with high permeability with K ≥ 10^−2^ cm/s. When the water level drops, the effect of the drainage well on the elimination of seepage force was unobvious. Similarly, in the landslide with slightly lower permeability with K < 10^−6^ cm/s, groundwater was difficult to be extracted and the control effect was relatively poor.

## Figures and Tables

**Table 1:** Hydrological and mechanical properties of the Shuping landslide.

Composition	Material	Saturated Conductivity *k*_s_ (m/day)	Porosity	Unit Weight γ (kN/m^3^)	Effective Cohesion *c*’ (kPa)	Effective Friction Angle *φ*’ (°)
Slide mass	Silty clay with gravel	3.90	0.39	20.3	/	/
Slip zone	Silty clay	2.98 × 10^−2^	0.3	/	25.7	20.4
Slide bed	Marlstone	1.47 × 10^−4^	0.2	/	/	/
